# *streammd*: fast low-memory duplicate marking using a Bloom filter

**DOI:** 10.1093/bioinformatics/btad181

**Published:** 2023-04-07

**Authors:** Conrad Leonard

**Affiliations:** Department of Genome Informatics, QIMR Berghofer Medical Research Institute, Herston, QLD 4006, Australia

## Abstract

**Summary:**

Identification of duplicate templates is a common preprocessing step in bulk sequence analysis; for large libraries, this can be resource intensive. Here, we present streammd: a fast, memory-efficient, single-pass duplicate marker operating on the principle of a Bloom filter. streammd closely reproduces outputs from Picard MarkDuplicates while being substantially faster, and requires much less memory than SAMBLASTER.

**Availability and implementation:**

streammd is a C++ program available from GitHub https://github.com/delocalizer/streammd under the MIT license.

## 1 Introduction

It is often desirable to identify duplicates in sequencing data, e.g. for library complexity estimation, or to omit them when the number of unique molecules is important, e.g. allele frequency calculation.

Duplicate identification is readily performed after alignment by grouping together templates located at the same genomic coordinates. A widely-used tool for this purpose is MarkDuplicates from https://broadinstitute.github.io/picard/ (Picard Toolkit 2019). A single-pass implementation is also possible: template ends are hashed as received and subsequent hits are identified as duplicates e.g. SAMBLASTER (Faust and Hall 2014); this has the advantage of allowing pipelined operation and avoiding at least one expensive write-read cycle. With conventional hash tables, the memory requirements of this approach are considerable for large libraries—a 60× coverage human whole genome contains ∼1B templates and the resulting hash structure is tens of GB—here we use instead a Bloom filter (Bloom 1970) to achieve fast streaming operation in a small memory footprint.

## 2 Implementation

streammd is implemented as a C++ program running in a single process. A Bloom filter is initialized with k=10 hash functions and a bit array sized to meet user-specified memory and false-positive requirements. Input is QNAME-grouped SAM records. Template ends are calculated from the primary alignments in each QNAME group and a signature hashed into the Bloom filter. If the signature is already present the output SAM record is marked as a duplicate: the 0x400 FLAG bit is set and the PG:Z:streammd tag is added. By default, an error is generated if the stored item count exceeds that at which the false-positive rate is theoretically reached; this behavior can be toggled with —allow-overcapacity. Metrics are written to file at the end of processing.

## 3 Methods

**Figure 1.**
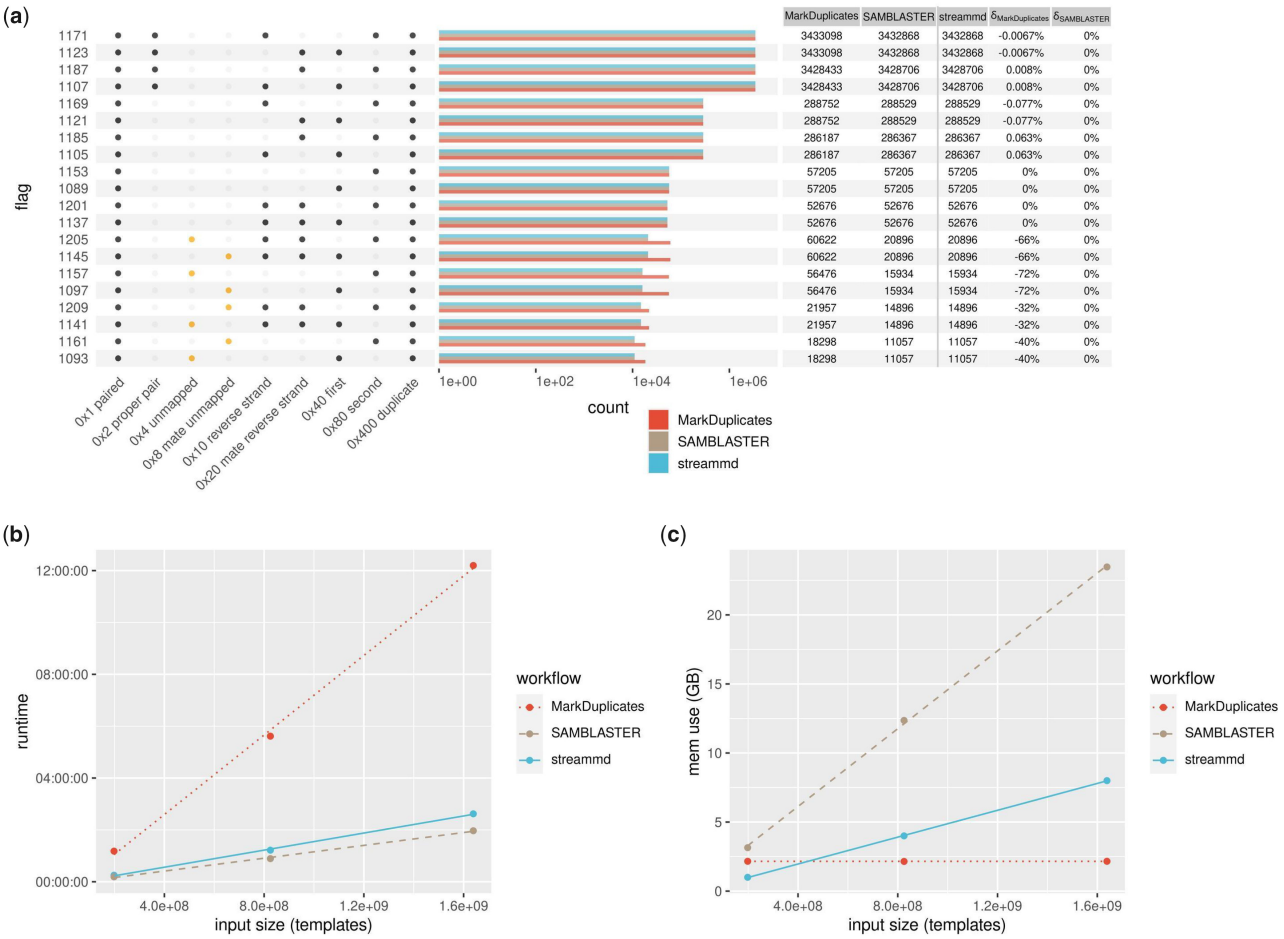
(a) Primary alignment duplicate flag counts after duplicate marking with MarkDuplicates, SAMBLASTER, and streammd. Input size = 3.97 × 10^8^ reads = 1.99 × 10^8^ templates. (b) MarkDuplicates, SAMBLASTER, and streammd runtime for three input sizes. (c) MarkDuplicates, SAMBLASTER, and streammd memory use for three input sizes. The small, medium, and large inputs correspond to single library paired-end human WGS bams of 30G, 117G, and 272G: ∼8×, 30×, and 60× read depth

In addition to extensive unit tests, to illustrate correctness we take Picard and SAMBLASTER as reference implementations and compare in Fig. 1a bulk duplicate FLAG counts in the outputs from MarkDuplicates, SAMBLASTER, and streammd run with default settings on *n* = 2 × 10^8^ templates. For this data using the default 4GiB memory setting for streammd yields a negligible expected Bloom filter false-positive rate of $<10^{-12}$.

In Fig. 1b and c, we show runtime and memory benchmarks for MarkDuplicates, SAMBLASTER, and streammd with different sized inputs. The small and medium inputs consist of 2 × 10^8^ templates and 8 × 10^8^ templates respectively from 2 × 101 bp Illumina sequencing of a single library from human lymphocyte sample MELA_0102 (Hayward et al. 2017); the large input consists of 1.6 × 10^9^ templates from 2 × 101 bp Illumina sequencing of a single library from human melanoma cell-line sample MELA_0114 (ibid.). These correspond to GRCh38 raw read depths of ∼8×, 30×, and 60×, respectively.

Latest versions of all tools were used—Picard Toolkit 2.27.4, SAMBLASTER 0.1.26, and streammd 4.2.1. Benchmarking was performed on a Dell c6320 with Intel^®^ Xeon^®^ CPU E5-2690 v4 processors and 256G RAM. MarkDuplicates was run using Java^®^ JRE 1.8.152, 2G heap space, serial GC, and output specified as SAM format to avoid compression overhead. streammd was run with the default maximum acceptable marginal false-positive rate of $10^{-6}$.

## 4 Discussion

All unit tests pass, including a suite of 47 tests ported from the MarkDuplicates codebase that demonstrate exact read-by-read equivalence of the two tools’ duplicate marking behavior for completely mapped templates. Empirically, in Fig. 1a, we observe that outputs from the three tools are generally highly concordant for bulk counts of duplicate flags. There are substantive differences between MarkDuplicates and the other two tools for counts of templates with one unmapped end. These are due to the duplicate detection algorithm of streammd and SAMBLASTER conforming to a stricter logic for these templates requiring identity of both the mapped end and mate unmapped status, than MarkDuplicates which requires only that the mapped end have a counterpart in some pair. There are also differences of a few ppm in counts of completely mapped templates (not discernible in the bar plot) between MarkDuplicates and the other two tools due to the input-order-dependent nature of streammd and SAMBLASTER duplicate assignment, in contrast to MarkDuplicates’ default best-quality strategy. The two approaches will in general yield different flag counts because duplicate sets frequently contain oppositely-oriented templates, which have different flag pairings. Notable in this case where expected Bloom filter false positives are zero is that the streammd and SAMBLASTER flag counts agree exactly.

In Fig. 1b, we observe that streammd averages ∼4.5× as fast as MarkDuplicates, as might be expected for single-pass operation. We also observe that SAMBLASTER averages ∼1.3× as fast as streammd, which is understandable as SAMBLASTER performs one hash and address operation per template where streammd performs 10. Profiling with gprof reveals that the majority of execution time in streammd is actually bit array access, a testament to the speed of the xxhash algorithm used here https://github.com/Cyan4973/xxHash (Collet 2012).

In Fig. 1c, we observe that streammd requires $\frac{1}{3}$ the memory of SAMBLASTER at all input sizes with the default maximum acceptable marginal false-positive rate of *P* = 10^−6^. For all inputs MarkDuplicates was run with the recommended 2G heap (Picard Toolkit 2019).

In Supplementary Fig. S2, we plot maximum and average CPU usage for the three tools. SAMBLASTER and streammd fully utilize a single core while MarkDuplicates averages 1.25 and peaks at over 4 due to JVM overhead.

Limitations of streammd due to streaming operation include inability to pick the “best” among duplicates or distinguish between optical and PCR duplicates, and loss of some unique sequence coverage due to Bloom filter false positives—although we believe the default setting of $10^{-6}$ is tolerable in most applications. The current implementation also accepts only SAM format inputs and requires a separate upstream tool to parse BAM or CRAM.

## 5 Conclusion

streammd achieves fast, accurate duplicate marking in a small memory footprint using the principle of a Bloom filter. Streaming operation and low memory use make it attractive for pipelined workflows, increasing the benefit of fast processing and allowing efficient packing of post-alignment tasks in HPC and container orchestration environments.

## Ethics

This research used previously published data for benchmarking. The QIMR Berghofer Human Research Ethics Committee approved use of public data (P2095).

## Supplementary Material

btad181_Supplementary_Data

## Data Availability

No new data were generated; sequence alignment files used in benchmarking are available from the European Genome-Phenome Archive (EGA) under dataset accession EGAD00001003388.
