# Supplementary figures and images for: streammd: fast low-memory duplicate marking using a Bloom filter
Source: Bioinformatics. 2023 Apr 7;39(4):btad181. doi: 10.1093/bioinformatics/btad181 (PMC10112951; doi:10.1093/bioinformatics/btad181)

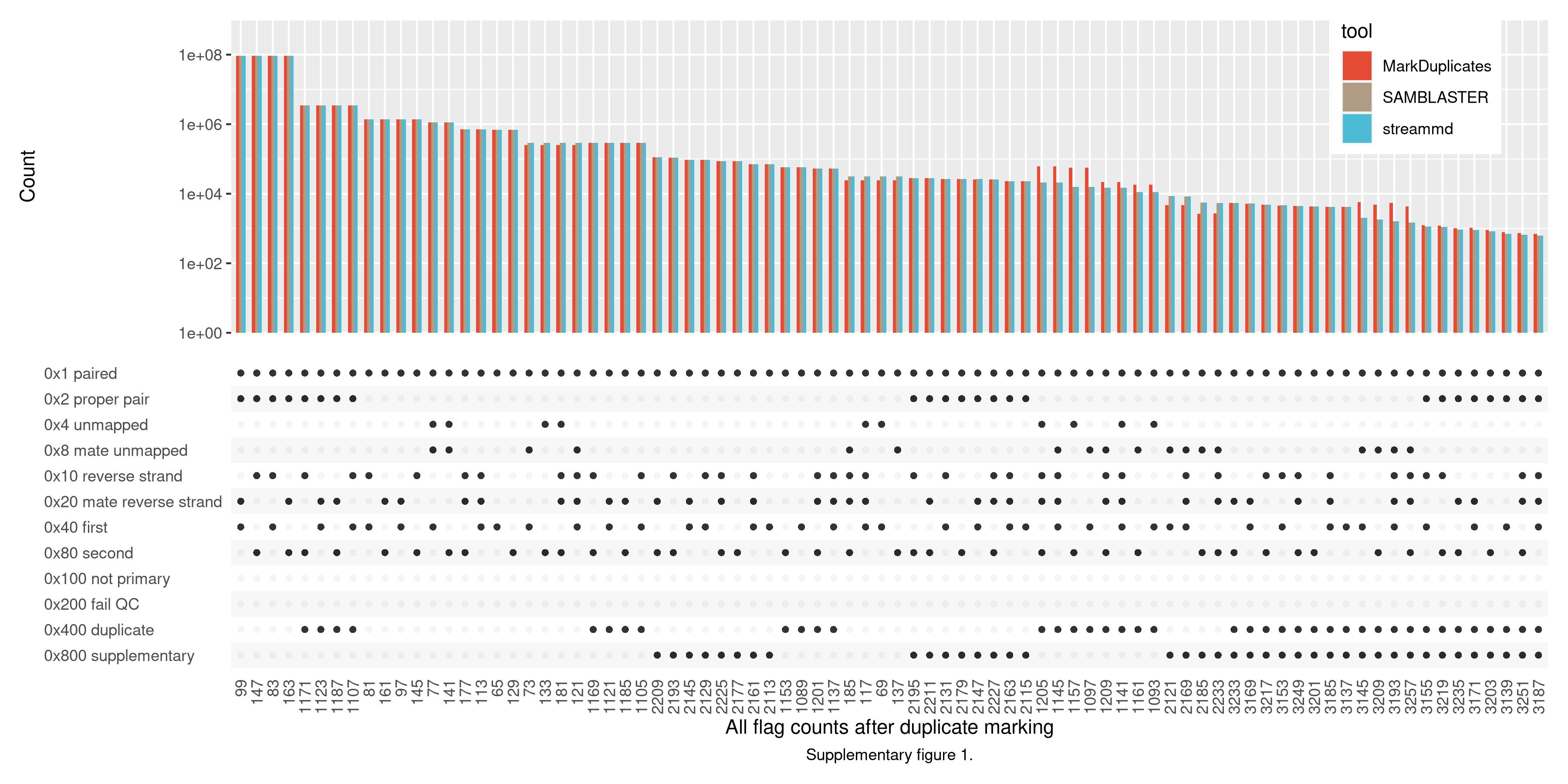

Supplement: btad181_Supplementary_Data [file btad181_supplementary_data.zip › Supplementary_figure_1.jpg]

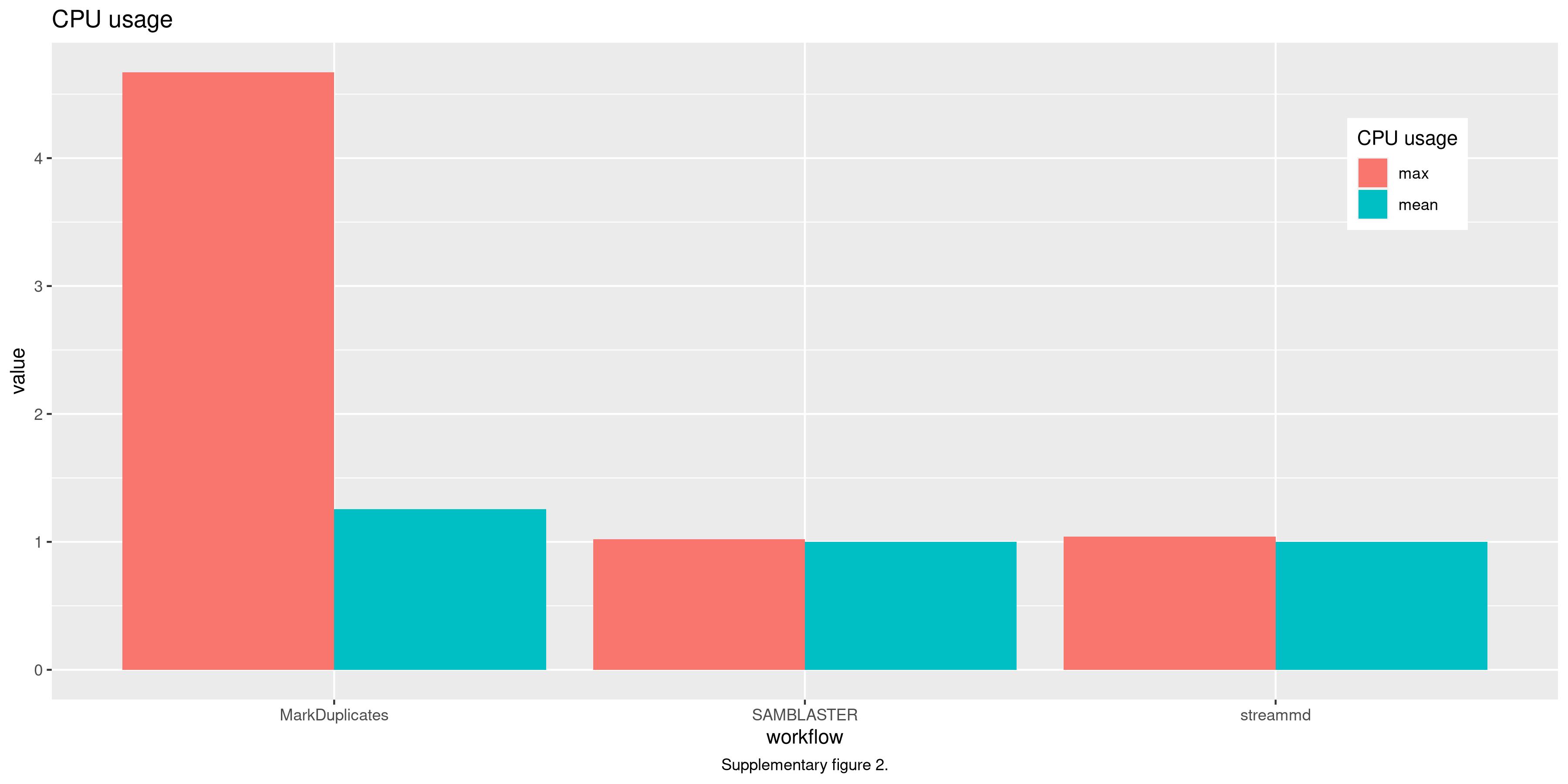

Supplement: btad181_Supplementary_Data [file btad181_supplementary_data.zip › Supplementary_figure_2.jpg]

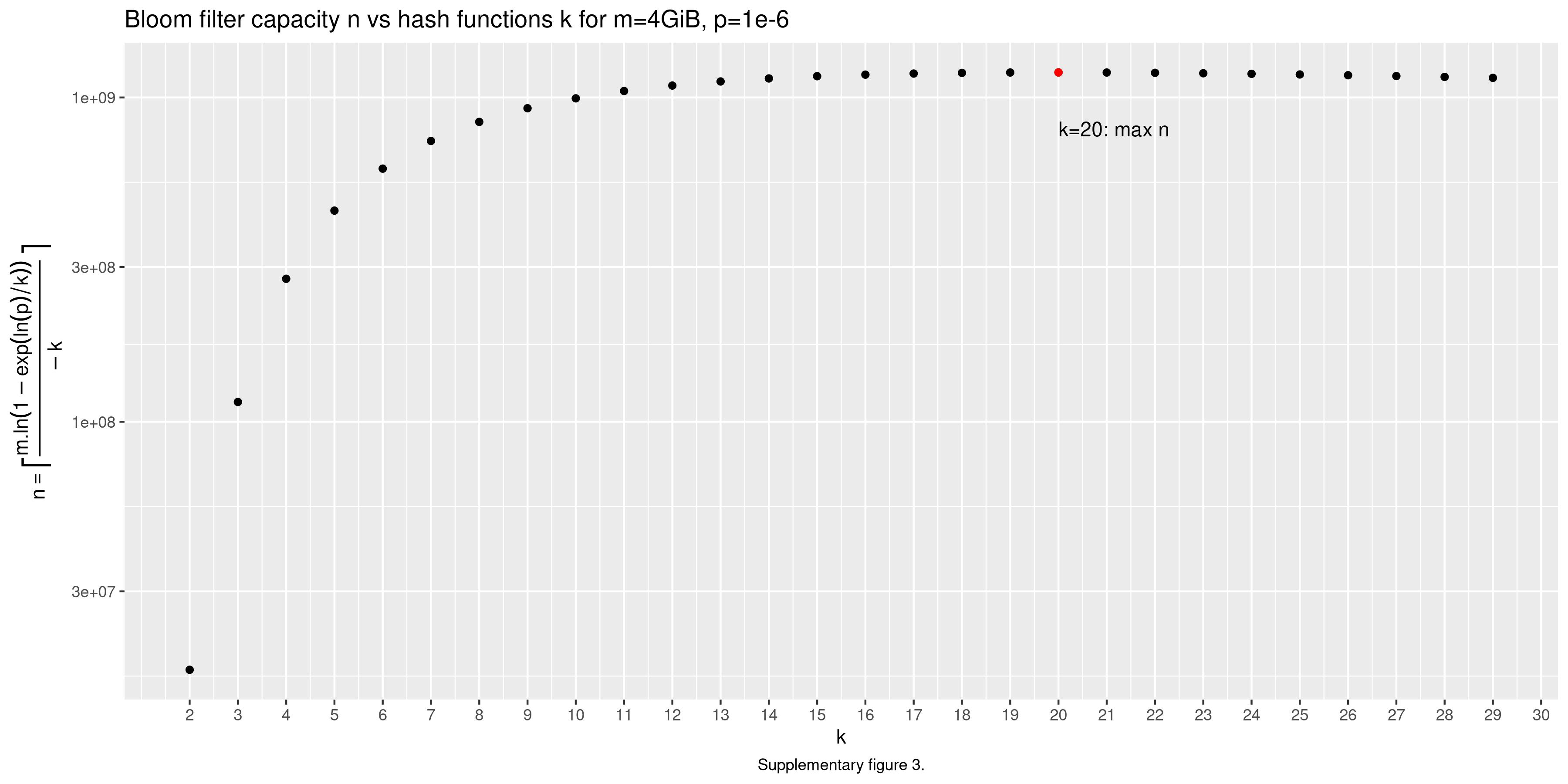

Supplement: btad181_Supplementary_Data [file btad181_supplementary_data.zip › Supplementary_figure_3.jpg]
